# Supplementary material for: Regulation of Fetal Genes by Transitions among RNA-Binding Proteins during Liver Development
Source: Int J Mol Sci. 2020 Dec 7;21(23):9319. doi: 10.3390/ijms21239319 (PMC7731027; doi:10.3390/ijms21239319)
Supplement: Supplementary file 1 [file ijms-21-09319-s001.pdf]

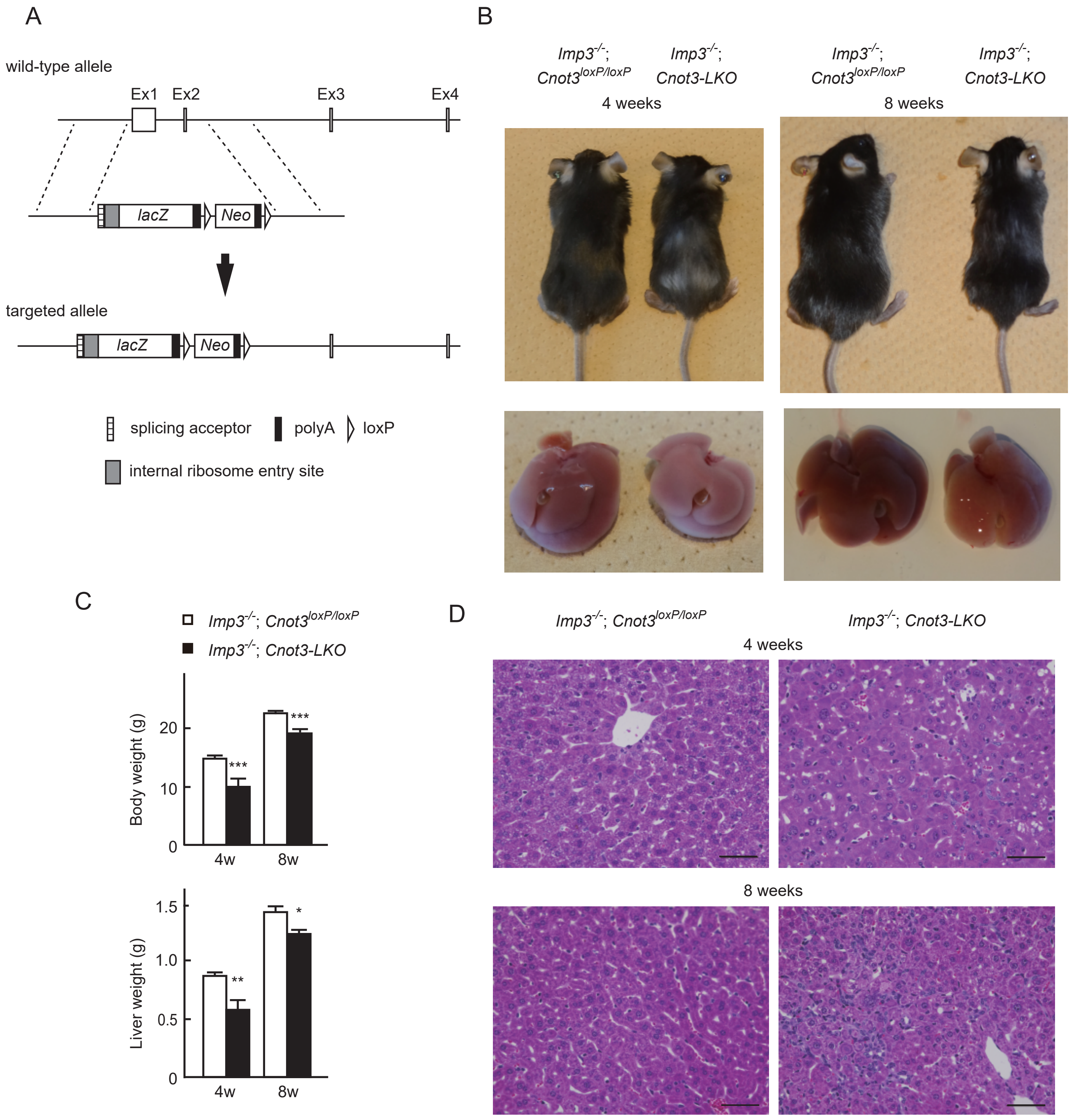

Supplementary Figure 1. Deletion of IMP3 does not influence the phenotype of *Cnot3-LKO* mice

(A) Schematic diagram of generation of the wild-type and *Imp3*-targeted alleles.

Exons (Ex) 1 and 2 were replaced with the *lacZ*-loxP-*neomycin resistant gene*-loxP cassette by homologous recombination.

(B) Appearances of whole body (upper) and liver (lower) of *Imp3*$^{-/-}$; *Cnot3*$^{loxP/loxP}$ and *Imp3*$^{-/-}$; *Cnot3-LKO* mice.

(C) Body (upper) and liver (lower) weights of *Imp3*$^{-/-}$; *Cnot3*$^{loxP/loxP}$ (4w: n=7, 8w: n=3) and *Imp3*$^{-/-}$; *Cnot3-LKO* mice (4w: n=3, 8w: n=4).

(D) Hematoxylin and eosin-stained livers from *Imp3*$^{-/-}$; *Cnot3*$^{loxP/loxP}$ and *Imp3*$^{-/-}$; *Cnot3-LKO* mice. scale bars: 50 μm.

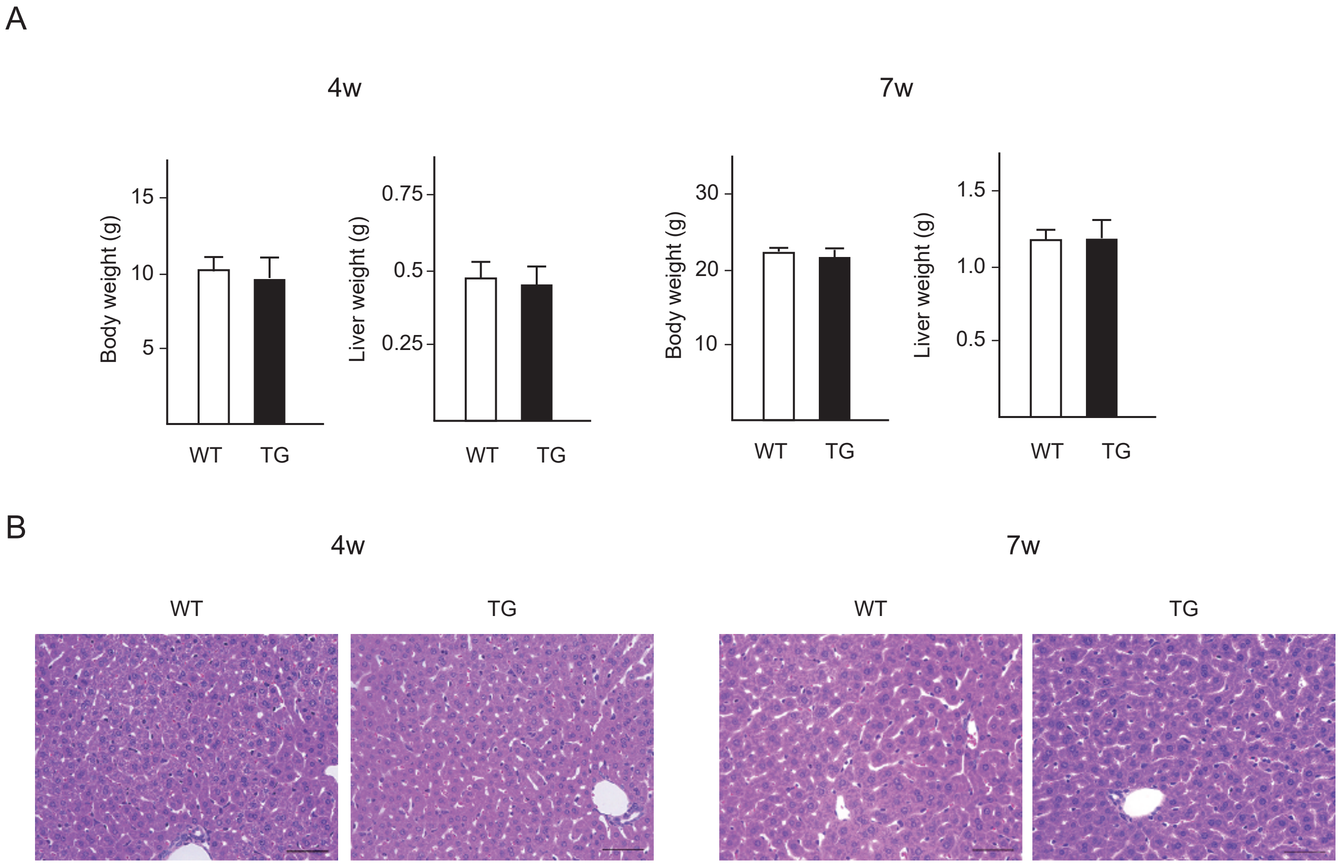

Supplementary Figure 2. Body weights, liver weights, and liver histology are comparable between wild-type and *Imp1*-transgenic mice
(A) Body (upper) and liver (lower) weights of wild-type (WT) (4w: n=6, 7w: n=4) and *Imp1*-transgenic (TG) mice. (4w: n=5, 7w: n=3).
(B) Hematoxylin and eosin-stained livers from WT and TG mice. scale bars: 50 μm.

Supplementary Table: primer lists

qPCR primers

gene	forward	reverse
Afp	catgctgcaaagctgacaa	ctttgcaatggatgctctctt
H19	cacttttcccaaagagctaacac	gctgggtagcaccatttctt
Igf2	accttcggccttgtggta	cgaaggccaaagagatgaga
Imp3	aaacagctttctcgctttgc	tccgcactttagcatctggt
unspliced Afp	gaacaggccgactgtagcat	ttcaggtccccttactacttgg
unspliced H19	agtgtctgtcccgctcgt	tgaagacatgagttaattgaacttgc
unspliced Igf2	cctgcccagtcctctactttt	ggaaggcccgaatttgtt
Gapdh	ctgcaccaccaactgcttag	gtcttctgggtggcagtgat

RNA-Immunoprecipitation RT-PCR primers

gene	forward	reverse
Afp	gcaggaattcggctatgcatcaccagtttt	gtgcctcgagcctaaggtatagaaatctca
H19	gtcagaattcggagccaagcctctaccccg	cgaactcgaggatggacccaggacctctgg
Igf2	acatctcgaggcaccctaaattacctgccc	cacggcggccgcaggtttgcgagcgttaacag

polyA tail assay primers

gene	gene specific fwd primer
Afp	gaccaggaagtctgtttcac
Igf2	gtgtttgcctcaactcagtc
H19	agtcccggagatagctttga
